# Younger age, medial meniscectomy and remnant‐sacrificing reconstruction techniques affect residual pivot shift after anterior cruciate ligament reconstruction using hamstring tendons

**DOI:** 10.1002/jeo2.70265

**Published:** 2025-05-12

**Authors:** Atsuo Nakamae, Eisaku Fujimoto, Mitsuhiro Nakamura, Tsuyoshi Takada, Yusuke Tsuyuguchi, Toshiya Kano, Akinori Nekomoto, Nobuo Adachi

**Affiliations:** ^1^ Department of Orthopaedic Surgery, Graduate School of Biomedical and Health Sciences Hiroshima University Hiroshima Japan; ^2^ Department of Orthopedic Surgery Chugoku Rosai Hospital Hiroshima Japan; ^3^ Department of Orhopaedic Surgery Hiroshima Prefectural Hospital Hiroshima Japan; ^4^ Department of Orthopaedic Surgery National Hospital Organization Kure Medical Center and Chugoku Cancer Center Kure Japan; ^5^ Department of Orhopaedic Surgery Mazda Hospital Hiroshima Japan; ^6^ Department of Orhopaedic Surgery Hiroshima City Hiroshima Citizens Hospital Hiroshima Japan

**Keywords:** anterior cruciate ligament, knee, multicentre study, pivot‐shift test

## Abstract

**Purpose:**

To investigate the factors affecting residual pivot shift after anterior cruciate ligament (ACL) reconstruction.

**Methods:**

This multicentre prospective cohort study included patients who underwent primary ACL reconstruction using an autologous hamstring tendon graft with or without the ACL remnant‐preserving technique. Multivariable logistic regression analysis was conducted to identify the factors that influenced residual pivot shift 1 year after ACL reconstruction. Age, sex, body mass index, interval between injury and surgery, preoperative pivot‐shift grade, side‐to‐side differences in anterior knee laxity, hyperextension, lateral and medial meniscus treatments, and ACL reconstruction procedure (remnant‐sacrificing single‐bundle reconstruction, remnant‐sacrificing double‐bundle reconstruction or remnant‐preserving single‐bundle reconstruction) were selected as independent variables in the multivariable model. Patients who underwent additional lateral extra‐articular tenodesis or anterolateral ligament reconstruction were excluded.

**Results:**

A total of 760 patients, including 394 males and 366 females (average age at surgery, 29.1 years; age range, 14–67 years), were enroled in this study. The postoperative side‐to‐side difference in anterior knee laxity was 0.8 ± 2.3 mm, and pathological positive results in the pivot‐shift test 1 year after ACL reconstruction were observed in 95 patients (12.5%). Factors that significantly affected postoperative positive pivot‐shift test results were younger age (odds ratio [OR] 1.71; *p* = 0.033), medial meniscectomy (OR, 3.48; *p* < 0.001), remnant‐sacrificing single‐bundle reconstruction (OR, 3.54; *p* < 0.001) and remnant‐sacrificing double‐bundle reconstruction (OR, 2.18; *p* = 0.034).

**Conclusions:**

Younger age, medial meniscectomy and remnant‐sacrificing ACL reconstruction techniques were associated with residual pivot shift 1 year after ACL reconstruction. Identifying risk factors for postoperative residual pivot‐shift is important for optimizing treatment decisions.

**Level of Evidence:**

Level II.

**Abbreviations**
ACL: anterior cruciate ligament
ALL: anterolateral ligament
IKDC: International Knee Documentation Committee
KOOS: Knee injury and Osteoarthritits Outcome Score
LET: lateral extra‐articular tenodesis
OR: odds ratio

## INTRODUCTION

The anterior cruciate ligament (ACL) is one of the most frequently injured knee ligaments, particularly in young individuals and active athletes. With recent advancements in the understanding of ACL anatomy and biomechanical function, the success rate of anatomic ACL reconstruction using autologous grafts has increased significantly, and a greater proportion of patients who undergo ACL reconstruction can resume sports activities at the pre‐injury level [9, 19]. However, residual rotatory laxity after ACL reconstruction, as demonstrated by a pathological positive result in the pivot‐shift test, remains an unresolved problem. Postoperative rotatory laxity appears to be correlated with poor clinical outcomes. Several studies have shown that the pivot‐shift test has significant associations with some subjective symptoms and functional outcomes after ACL reconstruction [3, 18]. Therefore, identifying factors associated with residual pivot shift after ACL reconstruction is important. Although several factors, including female sex, younger age, greater preoperative rotatory instability of the knee and lateral meniscectomy, have been suggested, the factors influencing postoperative rotatory laxity have not been well elucidated and remain a topic of debate [7, 8, 10, 12, 13, 16, 17, 20, 21, 32, 33, 36, 37, 39].

The majority of patients who undergo ACL reconstruction aim to return to sports activities 8–12 months after surgery. Therefore, postoperative rotatory knee stability 1 year after surgery is important for return to sports activity. Moreover, a grade 2 pivot‐shift can be considered a failure rather than just a limitation to return to sport at 1 year. The purpose of this multicentre prospective cohort study aimed to investigate the factors that affect residual pivot shift after ACL reconstruction. In the present study, the pivot‐shift test was performed while the patients were awake, as the postoperative pivot‐shift phenomenon was evaluated in outpatient clinics. Patients who underwent additional lateral extra‐articular tenodesis (LET) or anterolateral ligament (ALL) reconstruction were excluded from this study. Based on previous studies, we hypothesized that female sex, greater preoperative rotatory instability of the knee and lateral meniscectomy are significantly associated with a positive result in the postoperative pivot‐shift test.

## MATERIALS AND METHODS

### Patients

This study was approved by the Institutional Review Board of Hiroshima University Hospital (ID number: E2018‐1178) and the institutional review boards of all the participating hospitals. All the patients provided informed consent at their respective institutions. The Hiroshima Clinical ACL Research Project (Hiroshima CARP) is a prospective, longitudinal, multicentre cohort study designed to examine the prognosis after primary ACL reconstruction. Enrolment of patients at nine participating institutions began in January 2018. All patients had sustained an ACL tear diagnosed on the basis of clinical examination and magnetic resonance imaging (MRI) findings. Hiroshima University Hospital functioned as the data‐processing centre for the study and was responsible for storing all patient data from the included hospitals. In the current study, the inclusion criteria included patients who underwent primary ACL reconstruction using an autologous hamstring tendon graft between January 2018 and December 2020, and who were followed up for one year. Additional inclusion criteria required a normal contralateral knee, no history of surgery on the ipsilateral knee, and no posterior cruciate ligament insufficiency. Patients with grade III varus or valgus instability (with a valgus or varus opening at 0° and 30° of knee flexion) or congenital ACL deficiency were excluded from the study. Patients who underwent ACL reconstruction using a quadriceps autograft or bone‐patellar tendon‐bone autograft were also excluded, as were patients who underwent additional LET or ALL reconstruction. In addition, patients aged ≤13 years were excluded from this study because many of these patients underwent all‐epiphyseal or partial transphyseal techniques for ACL reconstruction. All surgeons who performed ACL reconstruction underwent a training programme for knee arthroscopy subspecialty at Hiroshima University Hospital for more than 4 years before participating in the study. At the university hospital, the surgeons were trained to perform knee surgeries and physical examinations, such as the pivot‐shift and the Lachman tests, in a standardized manner.

### Data sources and measurement of study variables

The patients completed an enrolment form that obtained information regarding their demographics, occupation, injury characteristics, sports participation level, interval between injury and surgery, activity level before injury and before surgery, knee surgical history (in either knee), and comorbidities. Validated patient‐reported outcome measures, including the Lysholm Knee Scale score [6], five subscale scores of the Knee injury and Osteoarthritis Outcome Score (KOOS) [31], and the International Knee Documentation Committee (IKDC) subjective form score [11], were used for preoperative evaluations. Participating surgeons were required to complete a uniform evaluation form for recording physical examination findings, including the range of motion of the knee, the pivot‐shift test grade, the Lachman test grade, and knee varus and valgus laxity. The standard clinical grading of the pivot‐shift test was determined by the participating surgeons in each hospital in accordance with the IKDC guidelines: 0 (equal), 1+ (glide), 2+ (clunk) or 3+ (gross). The Lachman test grade was assessed according to the IKDC criteria (grade 0 [negative] to 3 [severely abnormal]). The patient was placed in a supine position with the injured knee flexed to 20°–30°, and the injured leg was slightly externally rotated to relax the iliotibial band. These physical examinations were performed the day before surgery. Anterior knee laxity was measured for both knees using a Kneelax 3 Arthrometer (Monitored Rehabilitation Systems). The absolute values and side‐to‐side differences in anterior knee laxity were evaluated.

Immediately after each surgery, the operating surgeons were required to complete a uniform evaluation form to record the arthroscopic findings of the meniscus and articular cartilage. In addition, each surgeon completed a designated evaluation form outlining the details of the treatment, including the graft type for ACL reconstruction, graft size, length of the femoral tunnel, method of graft fixation, techniques used for femoral tunnel drilling, treatment of the meniscus and articular cartilage, and ACL reconstruction procedures (remnant‐sacrificing single‐bundle reconstruction, remnant‐sacrificing double‐bundle reconstruction, and remnant‐preserving single‐bundle reconstruction). For femoral bone tunnel drilling in ACL reconstruction, the far‐anteromedial portal technique was used in 64% of cases, the trans‐tibial technique in 19%, and the outside‐in technique in 17%. For femoral side fixation, fixed‐loop cortical suspension devices were used in 82% and adjustable‐loop devices in 14%. For tibial side fixation, the double‐stapling technique was used in 76% of cases. Interference screws were not used in this study. When arthroscopy revealed a continuous, thick ACL remnant between the intercondylar notch and tibia, the remnant was preserved, and remnant‐preserving single‐bundle ACL reconstruction (ACL augmentation) was performed. The positioning of the femoral and tibial bone tunnels was the same as in the single‐bundle ACL reconstruction. A quadrupled semitendinosus tendon was used to prepare a graft for the ACL augmentation procedure [23]. The meniscal condition or treatment was classified as normal, meniscal repair or partial meniscectomy.

Postoperative rehabilitation protocols were identical. After ACL reconstruction, patients were followed up periodically at the institutions where they underwent surgery. At the 1‐year postoperative on‐site visit, each patient completed the Lysholm Knee Scale, KOOS and IKDC subjective form. Activity was assessed using the Tegner activity scale 1 year after ACL reconstruction. In addition, the participating surgeons evaluated the anterior and rotatory knee laxities of the patients using the same protocol employed preoperatively. In the present study, grade 0 on the postoperative pivot‐shift test or no side‐to‐side difference in the postoperative pivot‐shift test grade was defined as a negative pivot‐shift test. A positive pivot‐shift test was defined in all other cases.

### Statistical analysis

Statistical analyses were performed using SPSS Statistics for Windows (version 22.0; IBM Corp.). Patient demographics and clinical characteristics were presented as means ± standard deviation, number of patients or frequencies (%). For normally distributed variables in independent groups, Student's independent *t*‐test was used to analyze the group distribution. Ordinal and non‐normally distributed variables were evaluated using the Mann–Whitney *U*‐test. The chi‐square test was used to compare the proportions among groups. Statistical significance was set at *p* < 0.05.

To identify potential predictors of residual pivot shift after ACL reconstruction, multivariable logistic regression with stepwise backward elimination was performed. Potential predictors included age at ACL reconstruction, sex, body mass index, interval between injury and surgery, knee extension angle before surgery, side‐to‐side differences in anterior knee laxity, pivot‐shift test grade before surgery, medial and lateral meniscal conditions or treatment (normal, meniscal repair or partial meniscectomy), and ACL reconstruction procedures (single‐bundle, double‐bundle or remnant‐preserving single‐bundle reconstruction). The included variables were based on previous studies. Parameter estimates were exponentiated to obtain odds ratios (ORs) and the corresponding 95% confidence intervals. When the age at reconstruction was included in a regression model, the variable was categorized as between 14 and 17 years, between 18 and 22 years or ≥23 years.

## RESULTS

In total, 760 patients (394 males and 366 females; average age at surgery, 29.1 years; age range, 14–67 years) were enroled in this study. Patient demographic data are listed in Table 1. Fifty‐two patients were aged >50 years. The mean postoperative side‐to‐side difference in anterior knee laxity was 0.8 ± 2.3 mm, and pathological positive results in the postoperative pivot‐shift test were found in 95 patients (12.5%) 1 year after ACL reconstruction.

**Table 1:** Patient characteristics.

Postoperative pivot shift	Residual pivot shift −	Residual pivot shift +	*p*‐value
Number of patients	665	95	−
Age (years)	29.4 ± 12.9	26.6 ± 12.3	**0.046**
Gender (male: female)	53.4%: 46.6%	41.1%: 58.9%	**0.016**
BMI at ACL reconstruction	24.1 ± 4.1	23.5 ± 3.5	0.128
Duration between injury and surgery (months)	16.6 ± 53.8	11.6 ± 39.2	0.382
Side‐to‐side difference of anterior knee laxity before ACL reconstruction (mm)	3.2 ± 2.5	3.8 ± 2.8	**0.036**
Knee extension angle before surgery (degree)	−1.2 ± 5.4	−0.9 ± 6.3	0.521
IKDC grading of the pivot shift test (Grade 0:1:2:3) before surgery	8%: 58%: 28%: 5%	16%: 46%: 26%: 12%	0.898
Tegner activity scale before injury	6.5 ± 1.7	6.5 ± 1.9	0.939
Lysholm knee scale before surgery	71.2 ± 19.2	74.3 ± 18.5	0.152
IKDC score before surgery	57.5 ± 17.3	58.3 ± 15.9	0.656
KOOS Symptom before surgery	74.1 ± 19.1	79.6 ± 18.6	**0.008**
KOOS Pain before surgery	76.2 ± 18.5	79.7 ± 18.0	0.083
KOOS ADL before surgery	85.4 ± 16.4	87.6 ± 16.1	0.227
KOOS Sports/Rec before surgery	48.6 ± 28.8	53.4 ± 26.0	0.124
KOOS QOL before surgery	47.2 ± 25.4	52.2 ± 23.3	0.073
ACL reconstruction procedures (single‐bundle reconstruction: double‐bundle reconstruction: remnant‐preserving single‐bundle reconstruction)	268(40%): 143 (22%): 254 (38%)	61 (64%): 17 (18%): 17 (18%)	**<0.001**
Condition or treatment of the meniscus (normal: partial meniscectomy: meniscus repair)			
Medial meniscus	468 (70%): 72 (11%): 125 (19%)	61 (64%): 23 (24%): 12 (13%)	**0.002**
Lateral meniscus	502 (75%): 84 (13%): 79 (12%)	69 (73%): 10 (11%): 16 (17%)	0.361

*Note*: Values are reported as mean ± SD, %, or number of patients. Bold values indicate statistical significance.

Abbreviations: ACL, anterior cruciate ligament; BMI, body mass index; IKDC, International Knee Documentation Committee; KOOS, knee injury and osteoarthritis outcome score.

Data on the clinical outcomes obtained 1 year postoperatively are listed in Table 2. Side‐to‐side difference of anterior knee laxity after ACL reconstruction was significantly higher in the residual pivot‐shift group than in the non‐residual pivot‐shift group (*p* < 0.001). There were no significant differences in the post‐operative scores (KOOS, IKDC score, Lysholm knee scale and Tegner activity scale) between the two groups.

**Table 2:** Clinical outcomes at 1 year postoperatively.

Postoperative pivot shift	Residual pivot shift −	Residual pivot shift +	*p*‐value
Side‐to‐side difference of anterior knee laxity after ACL reconstruction (mm)	0.6 ± 2.1	2.0 ± 2.9	**<0.001**
Tegner activity scale	5.4 ± 1.9	5.4 ± 2.0	0.986
Lysholm knee scale	93.5 ± 10.5	94.6 ± 7.4	0.285
IKDC score	81.8 ± 14.2	81.5 ± 14.2	0.841
KOOS Symptom	87.8 ± 13.5	89.7 ± 11.5	0.185
KOOS Pain	92.3 ± 10.0	93.7 ± 8.2	0.186
KOOS ADL	97.3 ± 5.2	97.5 ± 5.2	0.739
KOOS Sports/Rec	85.2 ± 17.1	85.3 ± 16.5	0.949
KOOS QOL	79.8 ± 19.0	82.5 ± 16.6	0.178

*Note*: Values are reported as mean ± SD or %. Bold values indicate statistical significance.

Abbreviations: ACL, anterior cruciate ligament; IKDC, International Knee Documentation Committee; KOOS, knee injury and osteoarthritis outcome score.

The results of the multivariable logistic regression analysis identifying risk factors for residual pivot shift after ACL reconstruction are summarized in Table 3. Significant factors affecting positive postoperative pivot‐shift test results included younger age (OR, 1.71; *p* = 0.033), medial meniscectomy (OR, 3.48; *p* < 0.001), remnant‐sacrificing single‐bundle reconstruction (OR, 3.54; *p* < 0.001), and remnant‐sacrificing double‐bundle reconstruction (OR, 2.18; *p* = 0.034).

**Table 3:** The factors that significantly influenced positive results in the postoperative pivot shift test.

	Crude OR (95% CI) (Univariable regression analysis)	*p*‐value	OR (95% CI) (Multivariable logistic regression)	*p*‐value
Age at ACLR				
14–17	1.67 (1.04–2.69)	**0.033**	**1.71** (**1.04**–**2.79)**	**0.033**
18–22	0.84 (0.49–1.79)	0.839		
≥23 (referent)	1			
Gender				
Male (referent)	1			
Female	1.64 (1.06–2.54)	**0.025**	1.48 (0.93–2.35)	0.101
BMI at ACLR	0.96 (0.90–1.01)	0.128		
Duration between injury and ACLR	1.00 (0.99–1.00)	0.387	0.99 (0.99–1.00)	0.078
Knee extension angle before ACLR	1.01 (0.97–1.06)	0.520		
Side‐to‐side difference in anterior knee laxity before ACLR (mm)	1.09 (1.01–1.19)	**0.036**		
Pivot shift test grade before ACLR (IKDC grading)	1.06 (0.79–1.42)	0.719		
Medial meniscal treatment				
Normal (referent)	1			
Partial meniscectomy	2.35 (1.36–4.06)	**0.002**	**3.48** (**1.90**–**6.36)**	**<0.001**
Meniscal repair	0.66 (0.34–1.29)	0.222		
Lateral meniscal treatment				
Normal (referent)	1			
Partial meniscectomy	0.88 (0.44–1.78)	0.722		
Meniscal repair	1.50 (0.83–2.71)	0.182		
ACLR procedure				
Remnant‐preserving single‐bundle reconstruction (referent)	1			
Single‐bundle reconstruction	3.40 (1.93–5.98)	**<0.001**	**3.54** (**1.99–6.29)**	**<0.001**
Double‐bundle reconstruction	1.78 (0.88–3.59)	0.109	**2.18** (**1.06–4.47)**	**0.034**

*Note*: Bolded values of OR (multivariable logistic regression) indicate statistically significant risk factors.

Abbreviations: ACLR, anterior cruciate ligament reconstruction; BMI, body mass index; CI, confidence interval; IKDC, International Knee Documentation Committee; OR, odds ratio.

## DISCUSSION

The most important finding of the present study was that the factors affecting pathological positive results in the pivot‐shift test after ACL reconstruction were younger age, medial meniscectomy and remnant‐sacrificing ACL reconstruction techniques. Regarding the reconstruction procedure, positive results in the postoperative pivot‐shift test were observed more frequently in patients who underwent remnant‐sacrificing single‐bundle ACL reconstruction (OR, 3.54; *p* < 0.001) and remnant‐sacrificing double‐bundle reconstruction (OR, 2.18; *p* = 0.034) compared to those who underwent the remnant‐preserving single‐bundle technique. Contrary to our hypothesis, female sex, a larger preoperative pivot‐shift grade and lateral meniscectomy were not risk factors for residual knee rotatory instability. Postoperative rotatory instability of the knee, indicated by a positive pivot‐shift test result, is widely known to significantly affect clinical outcomes and progression to knee osteoarthritis [3, 15, 18, 23]. The results of our multicentre study suggest that young patients with medial meniscal tears should be carefully evaluated preoperatively owing to the risk of residual pivot shift.

The clinical outcomes of ACL reconstruction have improved, and ACL reconstruction is well recognized as a promising procedure, especially in young and active patients with ACL injuries. Many favourable objective or subjective outcomes after ACL reconstruction with various techniques have been reported, as shown in meta‐analyses or systematic reviews [5, 14, 34]. However, residual rotatory laxity after ACL reconstruction, which correlates with poorer functional outcomes and satisfaction, remains an unresolved issue. Yasuda et al. [38] reported postoperative knee laxity after ACL reconstruction with hamstring tendons in previously published studies with a level of evidence of I or II. According to the study, although side‐to‐side differences in anterior knee laxity after ACL reconstruction improved from 1.0 to 1.4 mm, the negative pivot‐shift test ratio was 81.3%–97.1%. The negative pivot‐shift test ratio in the present study was 87.5%, comparable to the results of previous studies. A recent systematic review demonstrated that the frequency of grade II and III positive pivot‐shift tests was 6% after ACL reconstruction with anterolateral augmentation (LET or ALL reconstruction) and 14% after isolated ACL reconstruction [4]. The residual rotatory laxity demonstrated by positive results in the pivot‐shift test after ACL reconstruction correlates with poorer functional outcomes and satisfaction [3, 18]. In addition, a positive result in the pivot‐shift test predicts articular cartilage injury [24] and meniscal injury [7, 13, 21, 25]. Kocher et al. [18] reported that a high pivot‐shift grade was significantly associated with reduced patient satisfaction and sports participation and reduced knee functions such as difficulty cutting and twisting; however, instrumented knee laxity or Lachman examination had no significant relationships with any subjective symptoms and functions. Jonsson et al. [15] stated that a positive pivot‐shift test result after ACL reconstruction predicted osteoarthritis of the knee at 5–9 years after the reconstruction. Ayeni et al. [3] conducted a systematic review and reported that a positive pivot‐shift test result was significantly correlated with the final functional outcomes after ACL reconstruction in many randomized controlled studies. However, the present study showed that there were no significant differences in the post‐operative scores (KOOS, IKDC score, Lysholm knee scale and Tegner activity scale) between the residual pivot‐shift group and the non‐residual pivot‐shift group. A recent study concluded that although there was no association between the conventional IKDC grading of the pivot‐shift test in the awake condition and any patient‐reported outcomes postoperatively, patients' subjective apprehension during the pivot‐shift test after ACL reconstruction was significantly associated with the postoperative scores of the Tegner activity scale and three subscales of the KOOS [26]. Although many previous studies have evaluated postoperative pivot‐shift phenomenon in the awake condition in outpatient clinics, reliability of the pivot‐shift test while the patient was awake was likely limited owing to the occasional difficulty in evaluating an awake patient using the pivot‐shift test due to the patient's fear compared to the patient being under anaesthesia [27]. Further research on patients' subjective apprehension during the pivot‐shift test may be necessary in the future.

The sex‐related differences in residual rotational laxity after ACL reconstruction are controversial. Noojin et al. reported that female patients had greater graft laxity than male patients, as demonstrated by the KT‐1000 arthrometer, Lachman and pivot‐shift tests [28]. In addition, Salmon et al. demonstrated that female patients had significantly greater laxity than male patients in the Lachman, pivot‐shift and mean manual maximum tests at 1, 2 and 7 years postoperatively. However, no significant sex‐related differences were observed in self‐reported knee function, symptoms of instability or radiological findings [32]. In contrast, Tohyama et al. [35] reported no significant difference between male and female patients in the pivot‐shift test after anatomic double‐bundle ACL reconstruction. In their systematic review and meta‐analysis, Tan et al. demonstrated no significant sex‐related differences in most objective parameters, such as the Lachman and pivot‐shift test results, although subjective and functional results were worse in female patients [33]. Although generalized laxity, bone mineral density or hormonal factors in female patients have been considered as reasons for postoperative laxity after ACL reconstruction, we did not evaluate these factors in the patients included in this multicentre study. Further investigations are necessary to clarify these factors.

Preoperative rotatory and anterior knee laxity are likely factors that affect pathological positive results in the pivot‐shift test after ACL reconstruction. In a longitudinal multicentre cohort study, Ueki et al. reported that a greater preoperative pivot‐shift grade under anaesthesia was a risk factor for residual rotational laxity after ACL reconstruction [36]. Yamamoto et al. [37] used an intraoperative navigation system in ACL reconstruction, which can quantitatively examine knee laxity, and reported that anterior tibial translation during the Lachman test, preoperative pivot‐shift grade, posterior tibial reduction during the pivot‐shift test, younger age and meniscal injury at the time of surgery were significantly correlated with a higher risk of a positive postoperative pivot‐shift test result. Unlike previous studies, the preoperative pivot‐shift grade was not a risk factor for residual rotatory laxity in this study. One explanation is that although the pivot‐shift test was performed under anaesthesia in these previous studies, we conducted a pivot‐shift test in the awake condition, in which laxity may be underestimated. Unlike assessments performed in the awake state, pivot‐shift assessments under anaesthesia must be performed thoroughly and reliably because the patients do not show any muscle defence, which can cause underestimation of rotational laxity [27]. Moreover, although a large side‐to‐side difference in anterior knee laxity preoperatively was not a significant risk factor for residual rotational laxity in the multivariable logistic regression analysis, this study showed that the side‐to‐side difference in anterior knee laxity before surgery was significantly higher in the residual pivot‐shift group than in the non‐residual pivot‐shift group in the univariable regression analysis (Tables 1 and 3). Preoperative large anterior knee laxity could be a potential risk factor for residual rotational knee laxity.

Several previous studies have shown that lateral meniscal injury and its treatment are associated with the residual pivot‐shift test [7, 12, 16]. Conversely, in their multicentre study, Ueki et al. [36] concluded that the condition or treatment method of the lateral meniscus was not a significant risk factor for a residual pivot shift after ACL reconstruction. Magnussen et al. [20] showed that the presence of lateral and medial meniscal tears was associated with increased odds of a high‐grade pivot‐shift test before ACL reconstruction. In their study, the OR of medial meniscus tears (1.63) was higher than that of the lateral meniscus tears (1.41). In our multicentre study, medial meniscectomy was a significant risk factor for residual pivot shift after ACL reconstruction. Considering these studies together, medial meniscal injuries may predict residual pivot shift.

The final issue is the surgical procedure. Several studies have clarified the effectiveness of ACL reconstruction using the double‐bundle technique or ACL reconstruction with additional augmentation of the anterolateral structure to reduce rotational laxity [4, 5]. Notably, this multicentre study showed that ACL reconstruction with the remnant‐preserving technique [1, 22, 29] provided better results in the postoperative pivot‐shift test. The most recent meta‐analysis that compared clinical outcomes and knee stability in remnant‐preserving ACL reconstruction versus standard ACL reconstruction concluded that remnant‐preserving ACL reconstruction yields comparable clinical outcome scores and significantly improved knee stability relative to standard ACL reconstruction without remnant preservation, without increasing the complication rate [2]. Moreover, Okutan et al. reported that remnant preservation in the femoral and tibial tunnel apertures was an independent factor for graft healing 1 year after surgery [30]. The authors measured the signal‐to‐noise quotient (SNQ) value on MRI, which has been reported as a reliable method to assess graft healing. Lower SNQ values are associated with better biomechanical properties and well‐synovialised grafts. The authors concluded that aggressive remnant resection should be avoided to promote graft healing. Several potential advantages of remnant preservation, including preservation of the mechanoreceptors within the ACL remnant, enhancement of revascularisation and ligamentisation of the grafted tendon, and contribution of the remnant to knee stability, may have a positive effect on the anterolateral rotatory stability of the knee after surgery.

The present study had some limitations. First, both preoperative and postoperative pivot‐shift tests were performed while the patient was awake. A preoperative high‐grade pivot shift observed under anaesthesia may be a risk factor for residual pivot shift after surgery. Conversely, several studies have evaluated the postoperative grading of the pivot‐shift test under awake conditions. Second, this study only included patients who underwent ACL reconstruction using the hamstring tendons. Patients who underwent additional LET or ALL reconstruction were excluded. However, this unified surgical condition may have facilitated the identification of the true risk factors for residual pivot shifts after surgery. Third, anteromedial instability of the knee was not evaluated in this study. Fourth, using hamstring grafts for ACL reconstruction could be a potential risk factor for residual rotatory laxity. However, the optimal graft choice remains debatable. Fifth, patients with grade III varus or valgus knee instability were excluded from this study. In addition, there may be a potential effect of grade II varus or valgus injuries on rotatory laxity of the knee. Finally, the follow‐up period was too short to evaluate the clinical outcomes. However, most patients attempt to return to sports activities 8–12 months after ACL reconstruction. Therefore, the pivot‐shift phenomenon 1 year after surgery is important for return to sports.

From a clinical perspective, the findings of this study can aid in identifying patients at risk of residual pivot shift after ACL reconstruction, which is crucial for optimizing treatment strategies. Considering previous studies and the results of our study, when MRI shows medial meniscal injury in young patients, additional LET or ALL reconstruction may be considered to eliminate the pivot‐shift phenomenon after surgery. Female patients, a larger side‐to‐side difference in anterior knee laxity, and a greater pivot‐shift grade under anaesthesia preoperatively could be potential risk factors for residual pivot‐shift. Remnant‐preserving single‐bundle reconstruction is recommended if the ACL remnant can be preserved.

## CONCLUSIONS

Younger age, medial meniscectomy and remnant‐sacrificing ACL reconstruction techniques were associated with residual pivot shift 1 year after ACL reconstruction. Identifying risk factors for postoperative residual pivot‐shift is important for optimizing treatment decisions.

## AUTHOR CONTRIBUTIONS

Atsuo Nakamae and Nobuo Adachi were involved in concept creation, data collection, data analysis, construction of the first draft of the manuscript and editing of the final manuscript. Eisaku Fujimoto, Mitsuhiro Nakamura, Tsuyoshi Takada, Yusuke Tsuyuguchi, Toshiya Kano and Akinori Nekomoto were involved in concept creation and data collection.

## CONFLICT OF INTEREST STATEMENT

The authors declare no conflicts of interest.

## ETHICS STATEMENT

The current study was reviewed and approved by each participating site's respective institutional review board (Code; E2018‐1178), and informed consent was obtained from all participants included in this study.

## Data Availability

The data that support the findings of this study are available on request from the corresponding author, Atsuo Nakamae. The data are not publicly available due to restrictions on their content that could compromise the privacy of research participants.
